# Evaluation of benzocaine-based anesthetic gel in anuran skins extracts: A case study using the frog *Lithodytes lineatus* (Anura: Leptodactylidae)

**DOI:** 10.1371/journal.pone.0243654

**Published:** 2020-12-08

**Authors:** André de Lima Barros, Albertina Pimentel Lima, Maria Teresa Fachin-Espinar, Cecilia Veronica Nunez

**Affiliations:** 1 Departamento de Ecologia, Instituto Nacional de Pesquisas da Amazônia–INPA, Manaus, Amazonas, Brasil; 2 Laboratório de Bioprospecção e Biotecnologia—LABB, Instituto Nacional de Pesquisas da Amazônia–INPA, Manaus, Amazonas, Brasil; Universidade Federal de Minas Gerais, BRAZIL

## Abstract

Extracts made from the skin of dead *Lithodytes lineatus* frog individuals with the application of the benzocaine-based anesthetic gel, introduced into the oral cavity, were analyzed by ^1^H Nuclear Magnetic Resonance to investigate whether the application of this product (oral) can make studies that use extracts from the skins of these animals unfeasible. For comparison, we used skins of another species of anuran following the same death protocol. No trace of the benzocaine substance was found in the ^1^H-NMR spectra of the skin extracts from any of the tested anuran species. Still, using the hierarchical clustering model, it was possible to observe the formation of well-defined groups between the skin extracts of anurans and the anesthetic used to kill these animals. Our results suggest that the lethal dose of benzocaine in gel used inside the mouth of frogs may have no influence on potential results regarding the chemical composition or even bioassays using extracts made from the skin of these animals killed under this protocol since there was no detection of this substance for the analyzed samples.

## 1. Introduction

Studies involving analysis of the chemical composition of the cutaneous secretion of several species of anurans have been widely carried out, because it is possible to find a large number of substances with bioactive properties in the skin of these animals that present, mainly, great antimicrobial potentials [1–6]. There are several methods to extract the substances present in the body of frogs. One of the most used is the preparation of extracts made from the integument [7–9]. The method using skin extracts requires greater care during treatment in order to avoid indirect contamination of the samples during handling.

Currently, there are several debates about the use of some types of death protocols for anurans, such as, for example, cooling and freezing [10–12] since the perception of pain in these animals is still not well understood [13]. Thus, the use of anesthetics as a “humane” way to kill individuals in this taxonomic group has been recommended [7, 12, 14–17]. Benzocaine-based anesthetics are highly effective for both, anesthesia and death in amphibians, not requiring a large amount of the product to be able to kill them [12, 18–21]. However, recently, Saporito and Grant [22] found traces of the benzocaine substance coming from the Orajel^©^ (liquid) product in skin extracts made from individuals of the species *Melanophryniscus moreirae* and *Lithobates clamitans* killed with the oral application of this anesthetic and concluded that the application directly in the mouth in certain species of anurans, may invalidate potential studies on the chemical composition of the extracts of these animals, signalling false positives, such as inaccurate detection of substances and/or incorrect information about potential biological activities. The authors gave as an example the study by Amézquita et al. [23] who, through experiments, suggested that some populations of the frog *Allobates femoralis* showed higher toxicity in the extracts when used in mice and was considered by the authors to be from alkaloids present in the integument of this species. As *A*. *femoralis* belongs to a frog family that has no representative known for producing or sequestering diet alkaloids [24–28], this result was contested by Saporito and Grant [22] who described that the possible toxicity in *A*. *femoralis* is due to the presence of benzocaine substance that was converted to the animals' skin, detecting the presence of this substance experimentally using mass spectrometry (MS).

Studies showing the detection of substances used to kill anurans present in skin extracts of these animals are still scarce, but they are of great value since many experiments are conducted using different death protocols [12] and also, using anesthetics [7, 13–16].

In this study, we test whether the benzocaine used to kill individuals of the frog *Lithodytes lineatus* is transferred to the skin extracts of these animals.

## 2. Material and methods

### 2.1. Obtaining of the *Lithodytes lineatus* skin extracts and of the Benzotop^©^ product

Individuals of *L*. *lineatus* collected in three locations in the Brazilian Amazon (Cruzeiro do Sul [n = 3], Chandless State Park [n = 5] and Rio Branco [n = 3], all in the state of Acre), were killed using 40 mg of benzocaine-based anesthetic gel (Benzotop^©^ gel, DFL Indústria e Comércio S.A.) with direct application in the mouth to avoid contamination of the skins. Immediately after the deaths were confirmed the animals’ skins were removed through the inguinal incision from one end of the body to the other and sampled, by location, in microtubes containing methanol (100%) and prepared for extraction. Two substance extraction systems (10 mL / g of skin) were used based on differences in polarities between the solvents: 1) dichloromethane + methanol at a concentration 9:1 (v/v), and 2) methanol (100%). The use of different extracting solvents was made to be able to access different substances, potentially including, the benzocaine substance. Subsequently, in each extractor system, the skins were taken to an ultrasound bath (Unique^©^, model USC—1800, frequency 40Hz) for 20 minutes, and subsequently filtered on filter paper. This process was repeated three times, and the concentrates for each extraction, by location, packed in a single bottle. The extracts obtained were taken to the fume hood for total evaporation of the solvents.

Still, for comparison, we used skins of another species of anuran, *Adelphobates galactonotus* (n = 3), killed with the oral anesthetic application (based on benzocaine), however, disregarding the standardized amount (higher or lower) of the product used in *L*. *lineatus* individuals. The *A*. *galactonotus* skins used in this study were stored in the tissue bank of the Ecology Laboratory of the National Institute of Amazonian Research (INPA) and were used separately to assess whether there is an individual effect and / or the conservation time on the results. For analyzes that included this species of anuran, only methanolic extracts were used.

A sample containing 10 mg of the Benzotop^©^ gel product flavoured Pina Colada was solubilized using dichloromethane (DCM) and left to dry in a fume hood for total solvent evaporation. Subsequently, 10 mg of the methanolic extracts from the skins of individuals of *L*. *lineatus* and *A*. *galactonotus*, as well as the diluted sample of Benzotop^©^, were solubilized in deuterated chloroform (CDCl_3_) containing tetramethylsilane (TMS) as a reference solvent to be analyzed by the method Hydrogen Nuclear Magnetic Resonance spectroscopic (^1^H-NMR / NMR: Bruker, model Fourier 300, magnet 300 SB UltraShieldTM, 7.05T, 300 MHz).

**Fig 1:**
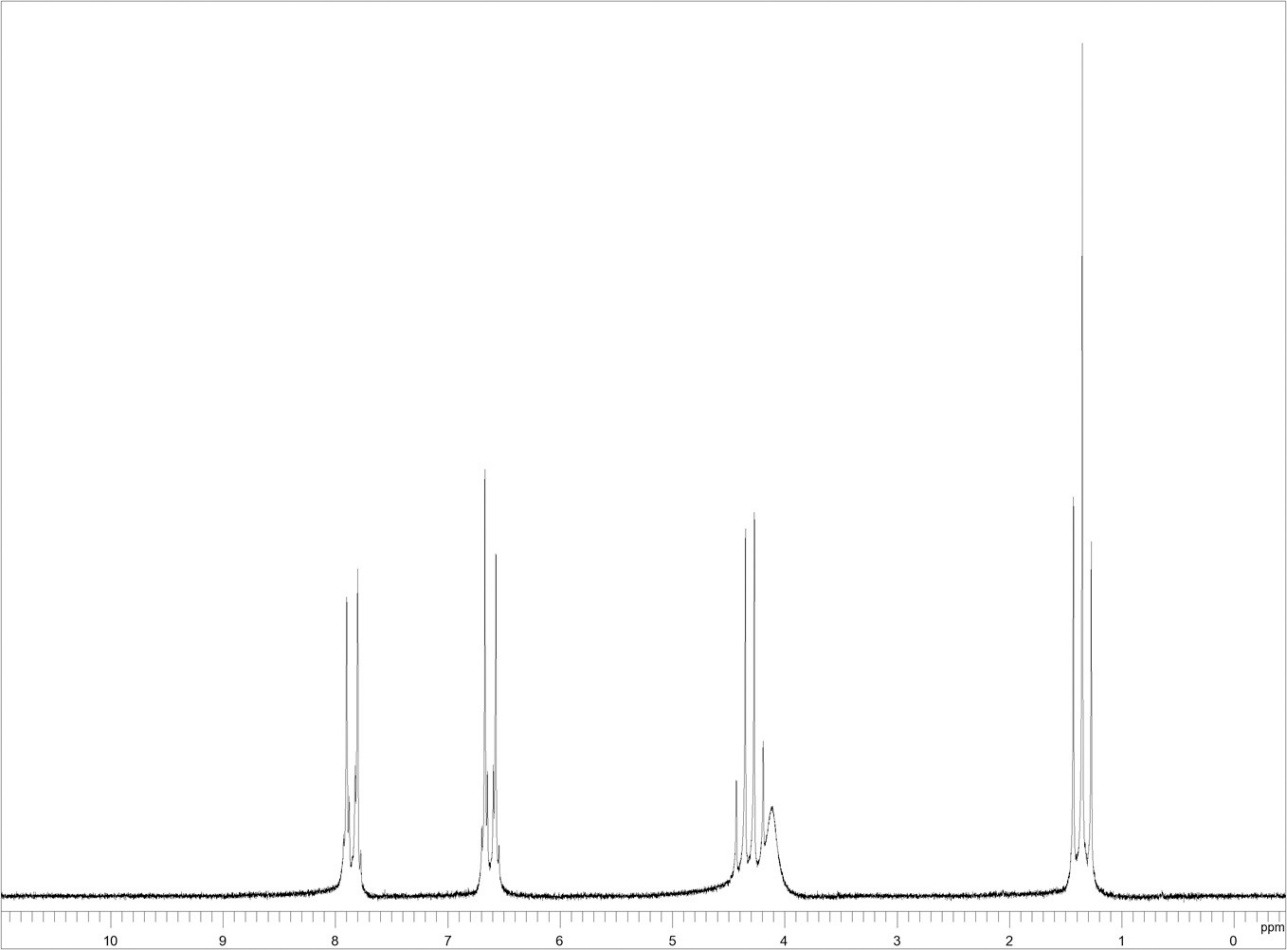
^1^H-NMR spectrum of the substance benzocaine solubilized in CDCl_3_. Withdrawal from and available at: https://scifinder.cas.org.

The search for the ^1^H-NMR spectrum of the benzocaine substance (standard) was performed in the *SciFinder* repository database (Fig 1). As benzocaine is a well-known substance, there was no difficulty in finding an available spectrum. Subsequently, the spectra referring to the tested samples were compared to the standard. All samples were solubilized in chloroform for ^1^H-NMR analysis and tetramethylsilane (TMS) was used as the reference solvent.

Mass spectrometry analysis–MS/MS, ESI+, 50 to 210 m/z amplification, positive mode (M+H)–was used to test the presence or absence of benzocaine substance in *L*. *lineatus* skin samples. The presence of benzocaine in the analysed samples is confirmed by detecting mass fragments of 166 m/z (benzocaine mass at positive mode) according to *SciFinder* repository database.

### 2.2. Chemometric analysis

The ^1^H-NMR spectra of extracts from frog’ skins and the Benzotop^©^ product had both phase distortions and baselines adjusted in the TopSpin program (version 4.0.7, Bruker Biospin) where they were automatically converted to CSV files (Comma-separated values). Subsequently, the CSV files were analysed using the R Studio program (R Studio, version 3.3 [29]). We evaluated potential differences or similarities between the samples tested, selecting specific regions in the ^1^H-NMR spectra to be excluded from the analyses, to perform a comparison based on the chemical shifts of interest, and eliminate potential noise from the sample (Table 1). In this analysis, only the regions corresponding to the chemical shifts of benzocaine were maintained. In total, five regions were chosen for exclusion.

**Table 1:** Regions of chemical shifts excluded from the chemometric analysis.

Number of cuts	Exclusion region (in ppm)
1	0.01–1.15
2	1.40–4.27
3	4.36–6.61
4	6.68–7.82
5	7.89–9.9

To highlight potential similarities or divergences between the different samples analysed, we used a Hierarchical Cluster Analysis (HCA) to assess whether there is the formation of groups between samples from Euclidean distances, the results of which are illustrated in a dendrogram. The analyzes were performed using the software R Studio.

### 2.3. Ethical approval

The National Institute of Amazonian Research approved the experiments with the frog species used in this study and the permissions for the use of animals were granted by the Ethics Committee for Research in the Use of Animals (CEUA) (INPA / CEUA, Protocol: 030 / 2014). The permissions for the animal collection were granted by the Chico Mendes Institute for Biodiversity Conservation (ICMBio / license number 57123–1). All experiments were carried out following the relevant guidelines and regulations.

## 3. Results

**Fig 2:**
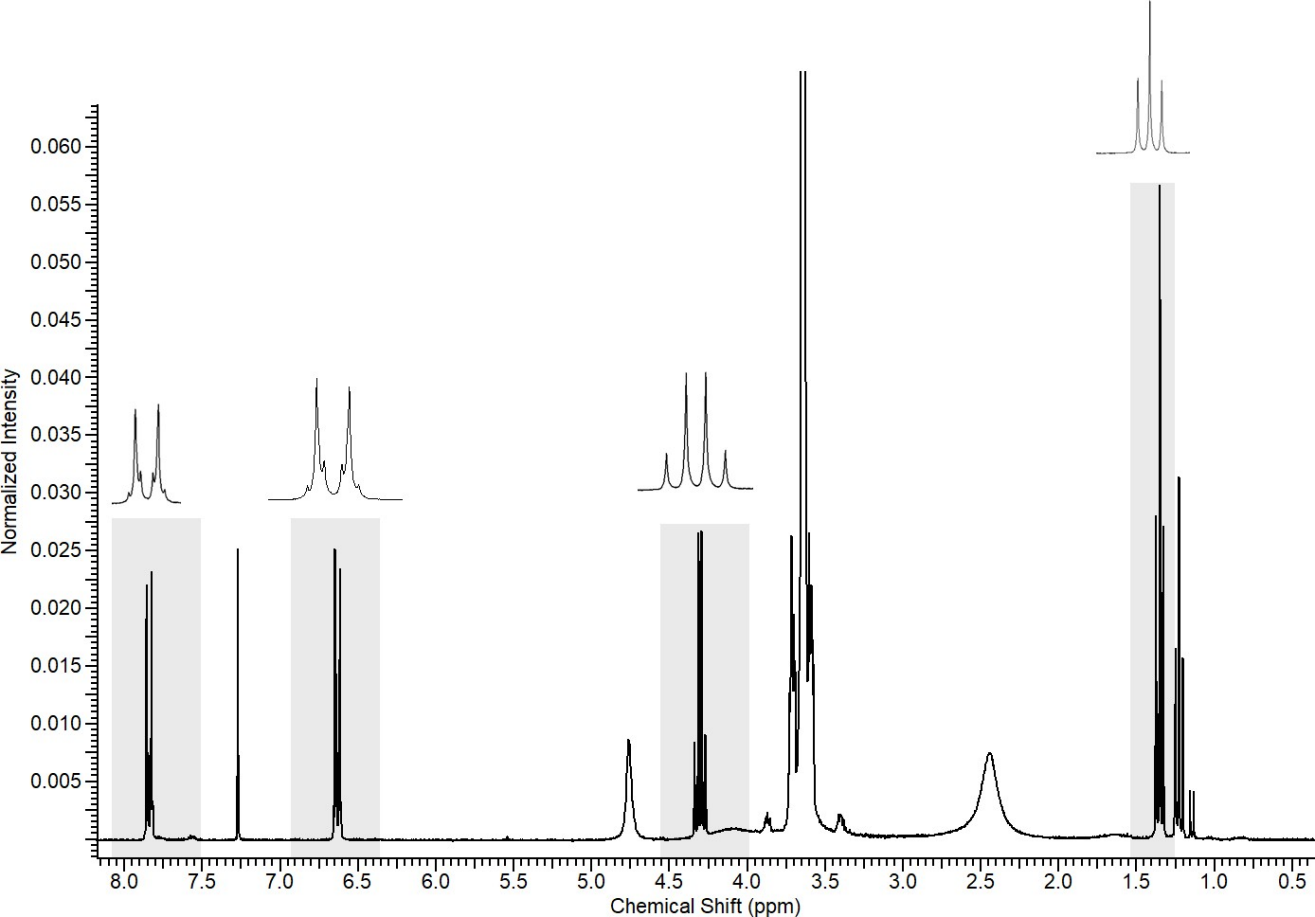
^1^H-NMR spectrum of Benzotop^©^ dichloromethanic extract. Gray rectangles describe the signals belonging to the substance benzocaine. Above, enlargements of the signals from the highlighted regions.

The signs referring to the benzocaine substance were detected in the sample made with the Benzotop^©^ anesthetic and are formed by regions with signs between I) 1.33 and 1.37 ppm; II) 4.27 and 4.37 ppm; III) 6.61 and 6.66 ppm, and IV) 7.83 and 7.87 ppm (Fig 2). The other signals detected in the spectrum come from other substances that are part of the general composition of the product, besides the solvent signal.

### 3.1. Comparative analysis between the ^1^H-NMR spectra of individuals of *Lithodytes lineatus* and the Benzotop^©^ product

**Fig 3:**
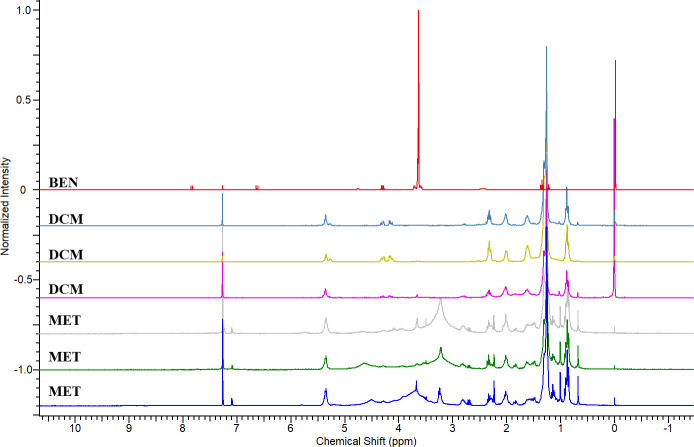
Overlapping ^1^H-NMR spectra of methanolic and dichloromethanic extracts from the skins of individuals of *Lithodytes lineatus* and the Benzotop^©^ product. In the spectrum: **BEN** corresponds to the extract of the Benzotop^©^ product; **DCM** corresponds to the dichloromethanic extracts of *L*. *lineatus*; **MET** corresponds to the methanolic extracts of *L*. *lineatus*.

The methanolic (**MET**) and dichloromethanic (**DCM**) extracts made with the skins of individuals of *Lithodytes lineatus* were compared with the dichloromethanic extract of the Benzotop^©^ (**BEN**) product to verify the presence or absence of characteristic signs of the benzocaine substance (Fig 3).

**Fig 4:**
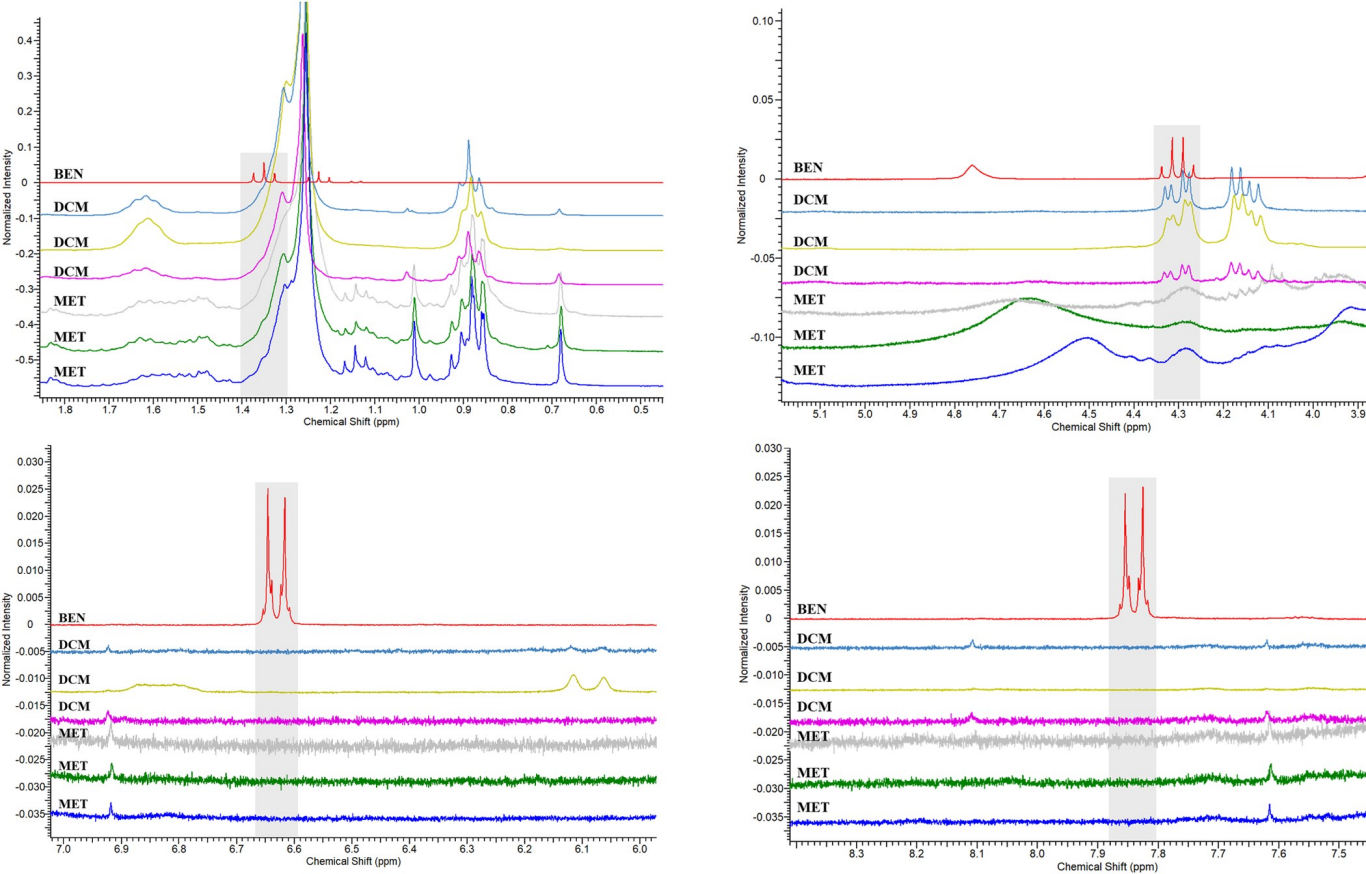
Magnification of the regions in the ^1^H-NMR spectra of methanolic and dichloromethanic extracts from the skins of *Lithodytes lineatus* individuals and the Benzotop^©^ product. In the spectrum: **BEN** corresponds to the extract of the Benzotop^©^ product; **DCM** corresponds to the dichloromethanic extracts of *L*. *lineatus*; **MET** corresponds to the methanolic extracts of *L*. *lineatus*. Gray rectangles describe the signals belonging to the benzocaine substance.

Through the analysis of the ^1^H-NMR spectra, it was possible to observe that **MET** presents a greater chemical complexity concerning **DCM**, clear by the superior amount of signals present in the spectra. Also, it was found that the benzocaine substance was not incorporated into the general composition of the skin extracts of *L*. *lineatus* individuals (Fig 4) killed with **BEN**, in neither of the two extractions, since the signs and consequently the characteristic chemical shifts of the substance in question were not detected.

**Fig 5:**
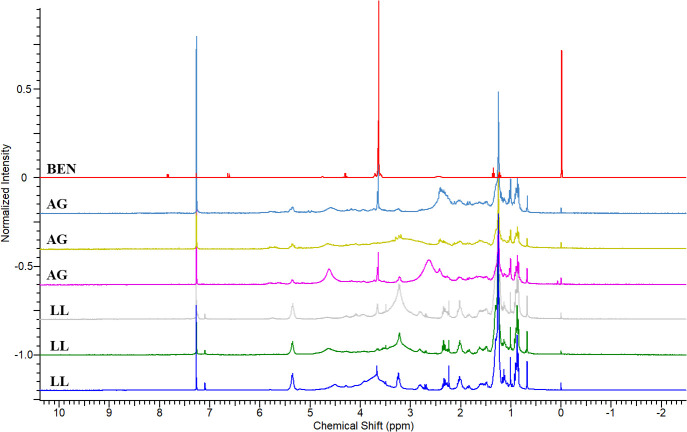
Overlapping ^1^H-NMR spectra of methanolic extracts from the skins of *Lithodytes lineatus* and *Adelphobates galactonotus* individuals and the Benzotop^©^ product. In the spectrum: **BEN** corresponds to the extract of the product Benzotop^©^; **LL** corresponds to *L*. *lineatus* extracts; **AG** corresponds to the extracts of *Adelphobates galactonotus*.

To validate the experiment, a comparison was made between BEN with extracts from the skin of another frog, *Adelphobates galactonotus* (**AG**), and the methanolic extracts from *L*. *lineatus* (**LL**) (Fig 5). The use of only *L*. *lineatus* methanolic extracts was due to the complexity of the samples compared to the dichloromethanic extracts, previously observed in the ^1^H-NMR spectra (Fig 4).

**Fig 6:**
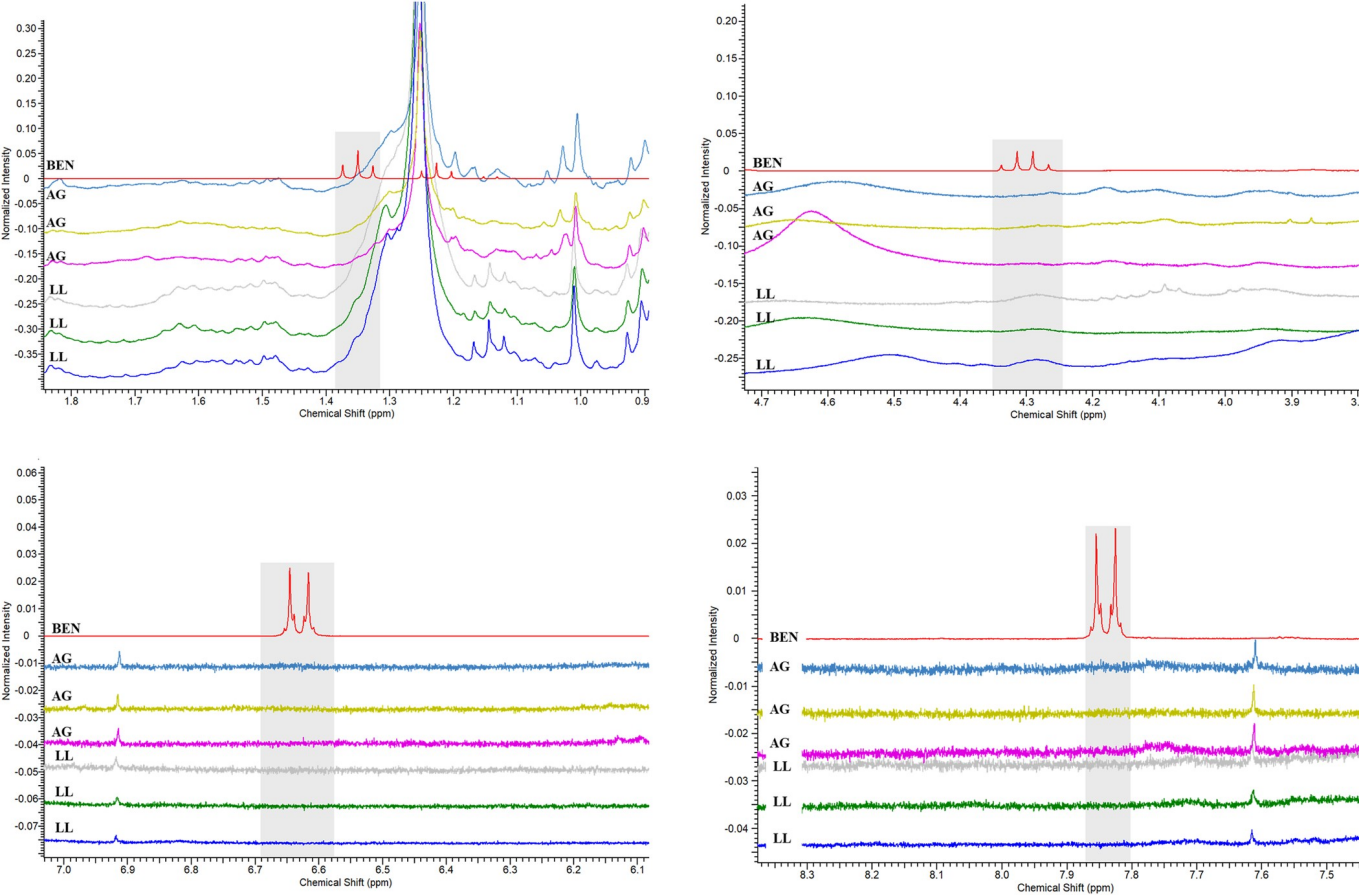
Magnification of the regions in the ^1^H-NMR spectra of the methanolic extracts of the skins of *Lithodytes lineatus* and *Adelphobates galactonotus* individuals and the dichloromethanic extract of the Benzotop^©^ product. In the spectrum: **BEN** corresponds to the extract of the Benzotop^©^ product; **AG** corresponds to the extracts of *A*. *galactonotus*; and **LL** corresponds to extracts of *L*. *lineatus*. Gray rectangles describe the signals belonging to the benzocaine substance.

There were also no signs of chemical shifts characteristic of the substance benzocaine in the ^1^H-NMR spectra of **AG** skin extracts. Thus, we found that there was no conversion of benzocaine to the skin extracts of either of the two tested species (Fig 6).

### 3.2. Hierarchical Cluster Analysis (HCA)

**Fig 7:**
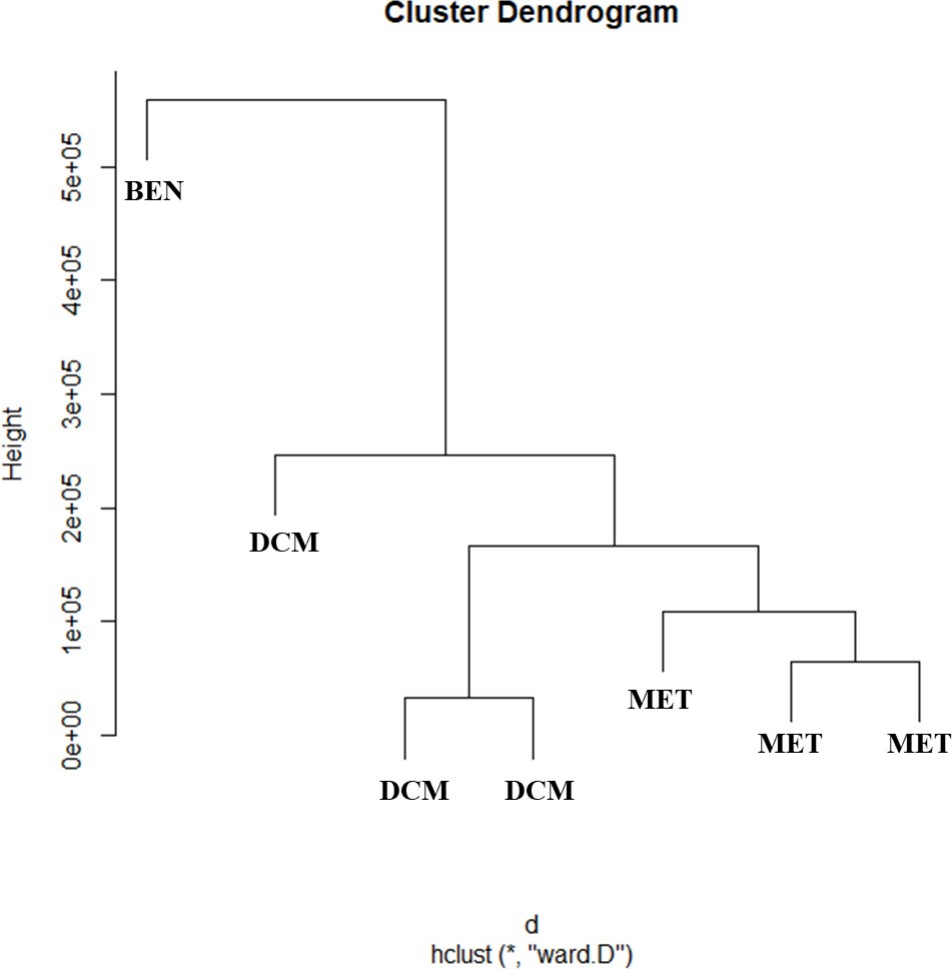
Cluster dendrogram of the spectra of methanolic and dichloromethanic extracts of *Lithodytes linetaus* and the Benzotop^©^ product. In the image: **BEN** = corresponds to the extract of Benzotop^©^; **DCM** = correspond to the dichloromethanic extracts of *L*. *lineatus*; **MET** = correspond to the methanolic extracts of *L*. *lineatus*.

The clusters analysis associated the samples by the similarity between the regions of interest (characteristic signs of the benzocaine substance). The formation of two well-defined clusters between the samples was evidenced, one formed by the skin extracts of *Lithodytes lineatus* (**LL**) individuals, in both extractions, and the other formed by Benzotop^©^ (**BEN**) (Fig 7).

**Fig 8:**
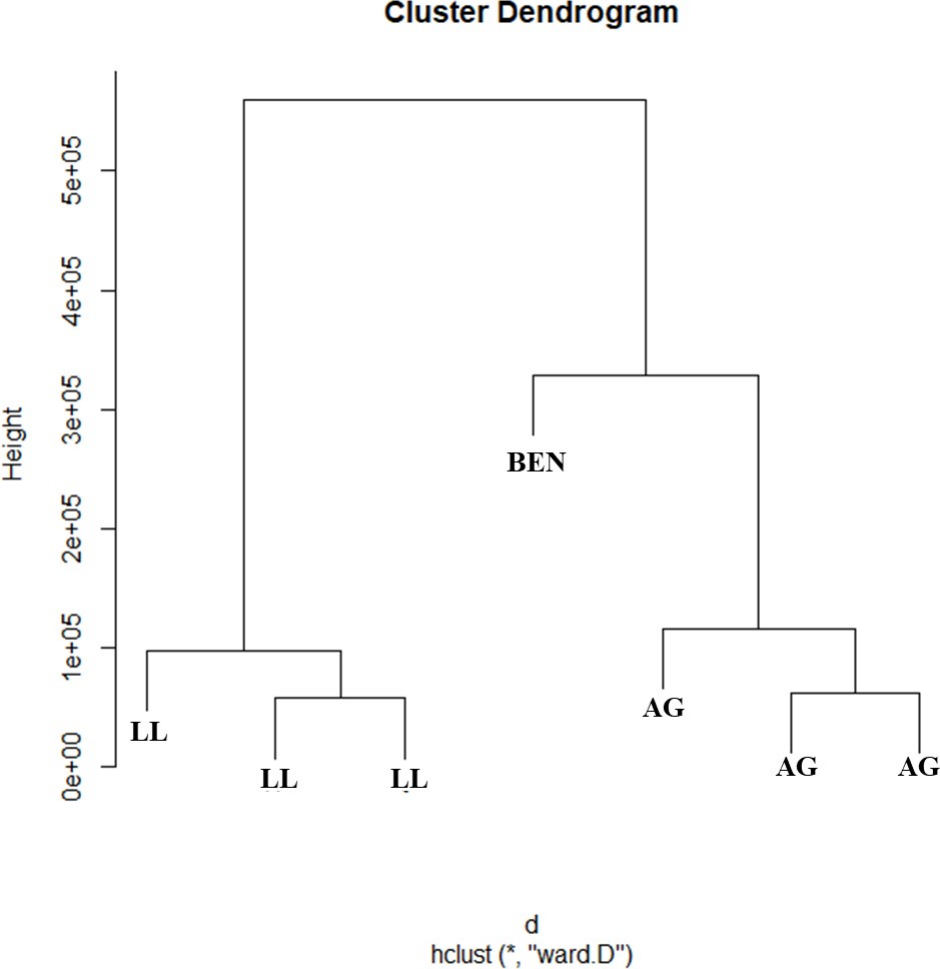
Cluster dendrogram of the spectra of methanolic extracts from the *Lithodytes linetaus* and *Adelphobates galactonotus* species and the dichloromethanic extract from the Benzotop^©^ product. In the image: **BEN** = corresponds to the Benzotop^©^ extract; **LL** = correspond to the *L*. *lineatus* extracts; **AG** = correspond to the *A*. *galactonotus* extracts.

Still, another analysis of clusters was made by adding **AG** and again showed that **BEN** formed a separate group in comparison to both samples, **AG** and **LL** (Fig 8). These results reinforce that one found in the analysis of the ^1^H-NMR spectra, where the presence of the benzocaine substance was not detected in any of the evaluated samples.

**Fig 9:**
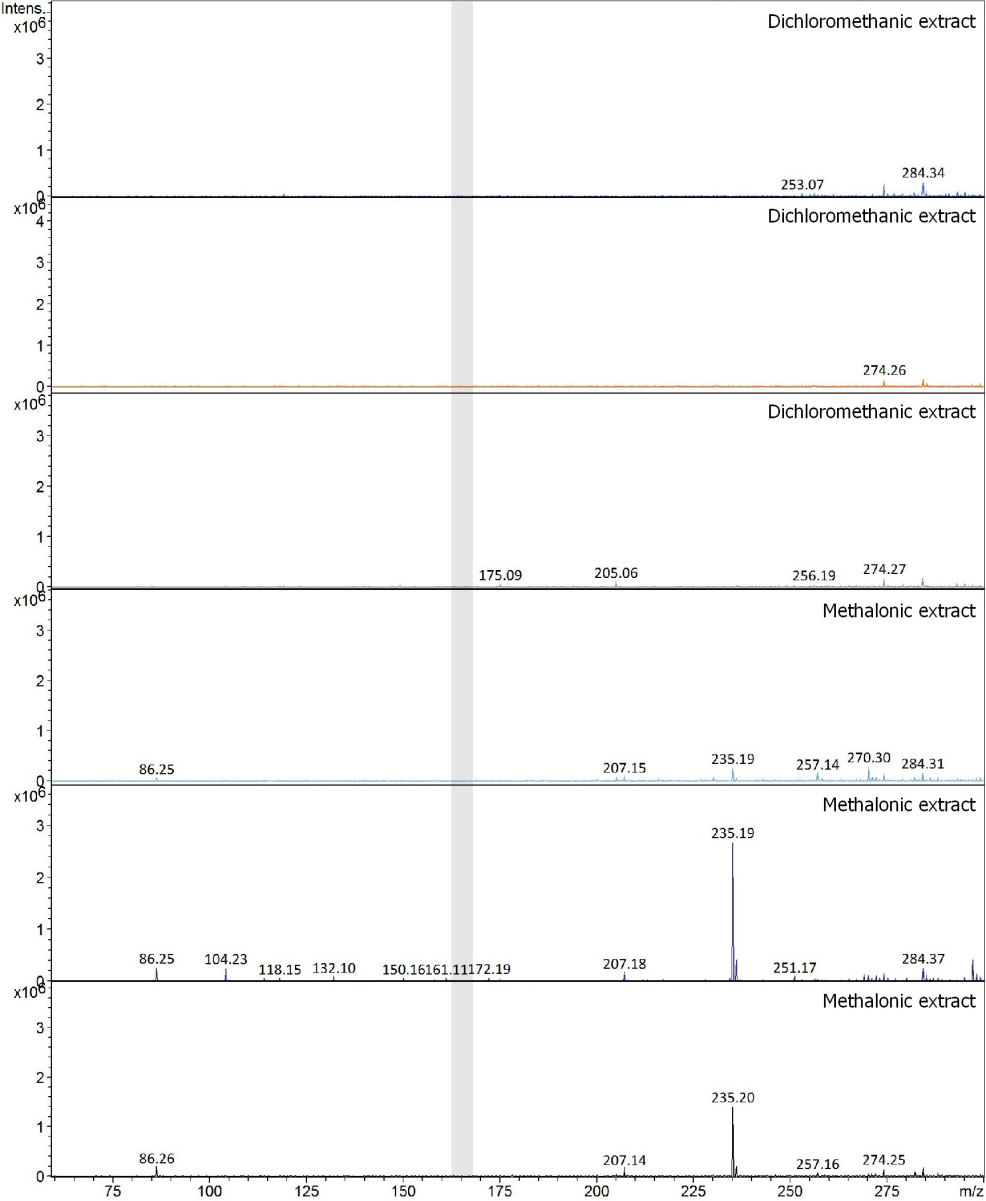
Mass spectra of the frog *Lithodytes lineatus* skin extracts (dichloromethanic and methanolic). Gray rectangles describe the region belonging to the benzocaine substance mass (166 m/z).

As well as verified by ^1^H NMR and Cluster Dendrogram analysis, benzocaine was not detected in *L*. *lineatus* skin extract samples by using mass spectroscopy analysis (Fig 9).

## 4. Discussion

The use of benzocaine-based anesthetics is considered a common practice and can be used for several purposes within herpetology. Kaiser and Green [30] observed that very low amounts of Orajel^©^ and Anbesol^©^ anesthetics applied to the heads of several anuran species cause temporary anesthesia, drastically reducing movement and can be used as a non-lethal method to facilitate “photographic tests” of these animals in field and laboratory. The use as an anuran death protocol is highly effective concerning lethality even when compared to other anesthetics [20, 21, 31, 32] besides to being considered a low-cost product and without restrictions on the purchase, facilitating access [12, 18]. On the other hand, the use of anesthetics to kill frogs that will subsequently have their skins removed for the preparation of extracts used in biological tests or investigation of the chemical composition present in the integument can be a problematic factor due to the potential contamination [22].

Some authors have used other anuran death protocols to avoid bias that can be caused by the use of anesthetics, such as, for example, cooling and freezing [9–11]. Despite eliminating the risk of skin contamination either by contact with the product or by the potential conversion of the anesthetic to the integument, the “cool and freeze” death protocol has generated many controversies in the scientific community, being considered by the American Association of Veterinary Medicine [12], as not humane and unacceptable.

The result of our study was contrary to that described by Saporito and Grant [22], in which large amounts of the benzocaine substance, of the product Orajel^©^ liquid, were found in the skins extracts of the *Melanophrynsicus moreirae* and *Lithobates clamitans* species. We found that in none of the extracts analyzed by ^1^H-NMR, of both species of anurans, the presence of the benzocaine substance from the anesthetic Benzotop^©^ in gel was detected, including in the skins (*Adelphobates galactonotus*) that were stored in tissue banks, whose lethal dosage used was indiscriminate. This allows us to infer that the use of Benzotop^©^ in gel introduced inside the mouth (lethal dose) for anurans does not prejudice possible studies regarding the chemical composition and/or biological activity of the extracts since the conversion to the skin was not evidenced.

We also suggest, based on the study by Saporito and Grant [22], that the use of benzocaine in liquid form should be avoided for studies involving the use of anuran skins extracts in bioassays since there is a possibility of signalling false positives; besides allowing inaccuracies as to the general chemical composition present in the extracts (mainly in the case of the use of crude extracts and not of isolated substances). We reinforce that other substances detection techniques (such as NMR and chemometrics) are important tools for better robustness and understanding of the results in these types of studies and that there is a need to investigate other parameters involving the interaction between the use of anesthetics and anurans, for example, whether sex, size, and age of these animals can influence the speed of conversion of these products to the integument.

## 5. Conclusion

Our results showed that the lethal dose of benzocaine in gel (Benzotop^©^ product) used inside the frogs' mouth was not converted to the integument. The benzocaine substance was not found in the extracts of both species of anurans evaluated, as evidenced by the ^1^H-NMR spectra. Also, in the Hierarchical Analysis of Clusters (HCA) it was possible to confirm the absence of benzocaine in the samples of the frogs' skin extracts.

## Supporting information

S1 File AG1_FID_CSV file: CSV file containing chemical shifts of the skin extract (DCM + MeOH) of *Adelphobates galactonotus* (specimen 01). (CSV)

S2 File AG2_FID_CSV file: CSV file containing chemical shifts of the skin extract (DCM + MeOH) of *Adelphobates galactonotus* (specimen 02). (CSV)

S3 File AG3_FID_CSV file: CSV file containing chemical shifts of the skin extract (DCM + MeOH) of *Adelphobates galactonotus* (specimen 03). (CSV)

S4 File LL1_DCM+MeOH_FID_CH_CSV file: CSV file containing chemical shifts of the dichloromethanic skin extracts from the frog *Lithodytes lineatus*. In the caption, CH corresponds to individuals collected at Chandless State Park.
(CSV)

S5 File LL2_DCM+MeOH_FID_CR_CSV file: CSV file containing chemical shifts of the dichloromethanic skin extracts from the frog *Lithodytes lineatus*. In the caption, CR corresponds to individuals collected in Cruzeiro do Sul municipality.
(CSV)

S6 File LL3_DCM+MeOH_FID_UFAC_CSV file: CSV file containing chemical shifts of the dichloromethanic skin extracts from the frog *Lithodytes lineatus*. In the caption, UFAC corresponds to individuals collected at the Federal University of Acre.
(CSV)

S7 File LL1_MeOH_FID_CH_CSV file: CSV file containing chemical shifts of the methanolic skin extracts from the frog *Lithodytes lineatus*. In the caption, CH corresponds to individuals collected at Chandless State Park.
(CSV)

S8 File LL2_MeOH_FID_CR_CSV file: CSV file containing chemical shifts of the methanolic skin extracts from the frog *Lithodytes lineatus*. In the caption, CR corresponds to individuals collected in Cruzeiro do Sul municipality.
(CSV)

S9 File LL3_MeOH_FID_UFAC_CSV file: CSV file containing chemical shifts of the methanolic skin extracts from the frog *Lithodytes lineatus*. In the caption, UFAC corresponds to individuals collected at the Federal University of Acre.
(CSV)
